# Stepwise radical cation Diels–Alder reaction via multiple pathways

**DOI:** 10.3762/bjoc.14.59

**Published:** 2018-03-27

**Authors:** Ryo Shimizu, Yohei Okada, Kazuhiro Chiba

**Affiliations:** 1 Department of Applied Biological Science, Tokyo University of Agriculture and Technology, 3-5-8 Saiwai-cho, Fuchu, Tokyo 183-8509, Japan; 2 Department of Chemical Engineering, Tokyo University of Agriculture and Technology, 2-24-16 Naka-cho, Koganei, Tokyo 184-8588, Japan

**Keywords:** Diels–Alder reaction, radical cation, rearrangement, single electron transfer, stepwise

## Abstract

Herein we disclose the radical cation Diels–Alder reaction of aryl vinyl ethers by electrocatalysis, which is triggered by an oxidative SET process. The reaction clearly proceeds in a stepwise fashion, which is a rare mechanism in this class. We also found that two distinctive pathways, including “direct” and “indirect”, are possible to construct the Diels–Alder adduct.

## Introduction

Umpolung, also known as polarity inversion, is a powerful approach in synthetic organic chemistry to trigger reactions that are otherwise difficult or impossible. In an umpolung reaction, the normal reactivity of the molecules being studied is reversed, e.g., electrophilicity is generated from a nucleophile. The single electron transfer (SET) process has been recognized as the most straightforward way to induce umpolung, which has recently been carried out by means of photo- [1–6] and electrochemical [7–12] approaches. A typical example involves an oxidative SET of an electron-rich and thus nucleophilic C–C double bond. The oxidative SET produces a radical cation species, which offers electrophilic reactivity for subsequent transformations. Enol ether radical cations are among the simplest members of this class and thus have been widely used in synthetic organic chemistry [13–15].

**Scheme 1:**
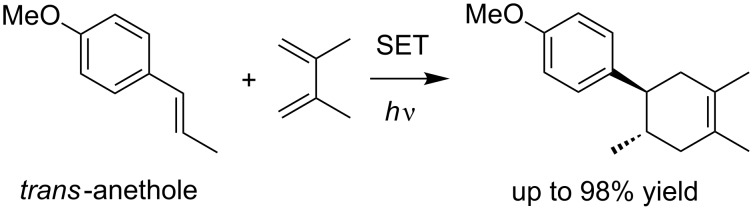
Radical cation Diels–Alder reaction of *trans*-anethole [17].

**Scheme 2:**
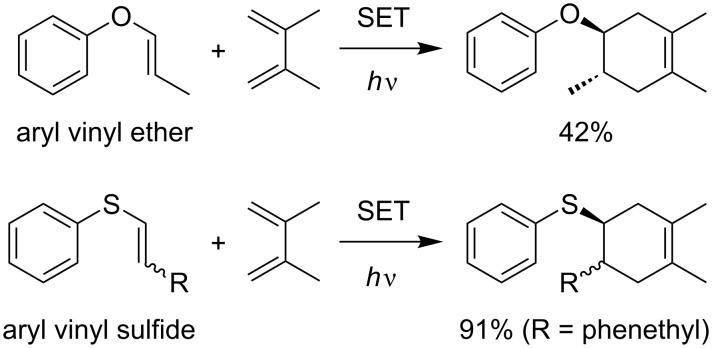
Radical cation Diels–Alder reactions of aryl vinyl ether and sulfides [17,25].

The Diels–Alder reaction is a classic reaction, and one of the most powerful methods to construct six-membered ring systems. A “normal” Diels–Alder reaction must consider an electronic matching of the substrates, with an electron-rich diene and electron-deficient dienophile as the general combination. Although having both an electron-rich diene and dienophile is a less- or non-effective combination, oxidative SET has proven to be able to overcome this mismatch and such a strategy is well-known as a radical cation Diels–Alder reaction. The use of *trans*-anethole as a model electron-rich dienophile is the representative example in this class. It was first reported by Bauld in 1986 [16] and was elegantly revisited by Yoon in 2011 [17] in the field of photoredox catalysis (Scheme 1). The reaction was further studied by Ferreira and Shores [18], followed by a unique mechanistic investigation by Rappé [19]. Although most recent examples of the radical cation Diels–Alder reaction have employed styrenes, the scope is not limited to such electron-rich dienophiles. Bauld and Yoon demonstrated that aryl vinyl ethers, enol ether equivalents, and aryl vinyl sulfides are also promising dienophiles for the reactions (Scheme 2) [17,20–25].

We have been developing oxidative SET-triggered cycloadditions of enol ethers by electrocatalysis [26–32] in lithium perchlorate/nitromethane electrolyte solution [33]. The reactions involve a radical cation chain process and are complete using a catalytic amount of electricity. During the course of our study, we discovered that radical cation Diels–Alder reactions are also possible by electrocatalysis, however, the scope was limited to styrenes [34]. Described herein is a stepwise radical cation Diels–Alder reaction of enol ethers by electrocatalysis, which proceeds via multiple unique pathways.

## Results and Discussion

**Scheme 3:**
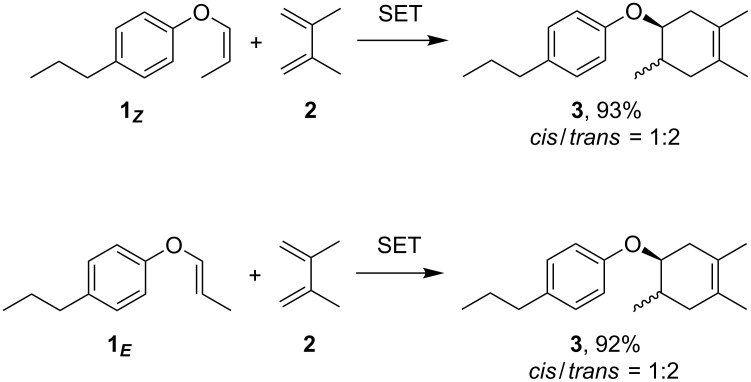
Radical cation Diels–Alder reaction of aryl vinyl ether (**1**). Conditions: 1.0 M LiClO_4_/CH_3_NO_2_, carbon felt electrodes, 1.2 V vs Ag/AgCl, 0.7 F/mol.

The present work began with the synthesis of the aryl vinyl ether **1** from *p*-propylphenol in 2 steps (Scheme S1 and Figure S1 in Supporting Information File 1). Both *E*- and *Z*-forms were readily purified by silica gel column chromatography. When the anodic oxidation of the *Z*-form **1*_Z_*** was carried out in the presence of 2,3-dimethyl-1,3-butadiene (**2**), the corresponding Diels–Alder adduct **3** was obtained in excellent yield (Scheme 3). The reaction was completed using a catalytic amount of electricity and therefore should involve a radical cation chain process. Since Diels–Alder adduct **3** was an approximately *cis*/*trans* = 1:2 mixture, the reaction must proceed in a stepwise fashion. This stereochemistry was confirmed when the *E*-form **1*_E_*** also gave the same synthetic outcome. Namely, the Diels–Alder adduct **3** was obtained as an approximately *cis*/*trans* = 1:2 mixture, indicating that the reaction was indeed stepwise.

**Scheme 4:**
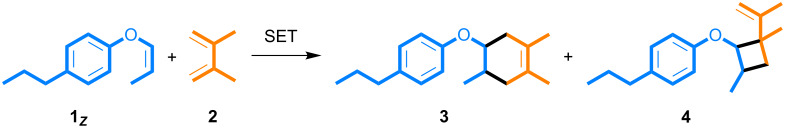
Oxidative SET-triggered reaction of aryl vinyl ether **1*_c_***. Conditions: 1.0 M LiClO_4_/CH_3_NO_2_, carbon felt electrodes, 1.2 V vs Ag/AgCl, 0.1 F/mol.

**Figure 1:**
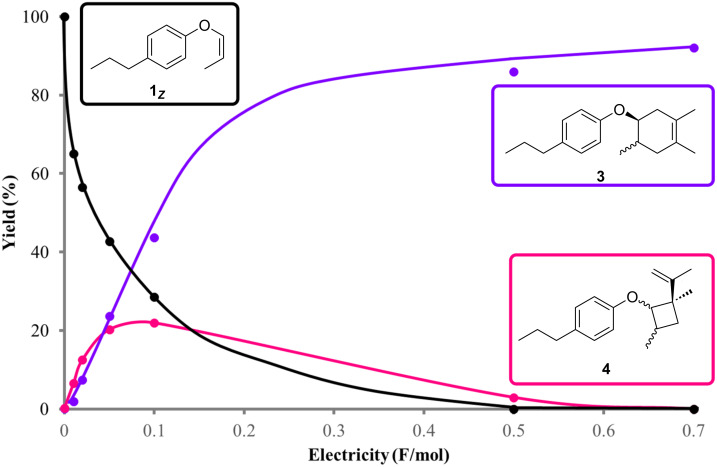
GC–MS Monitoring of the oxidative SET-triggered reaction of aryl vinyl ether **1*_c_***.

**Scheme 5:**
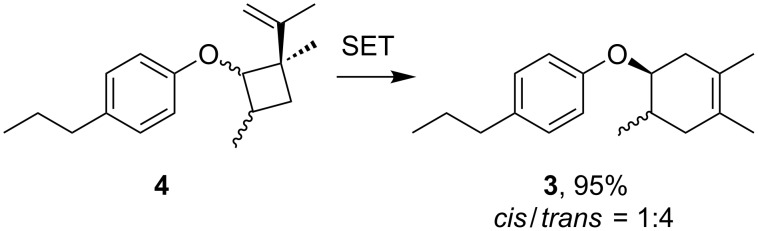
Oxidative SET-triggered rearrangement of vinyl cyclobutane **4**. Conditions: 1.0 M LiClO_4_/CH_3_NO_2_, carbon felt electrodes, 1.2 V vs Ag/AgCl, 0.5 F/mol.

**Scheme 6:**
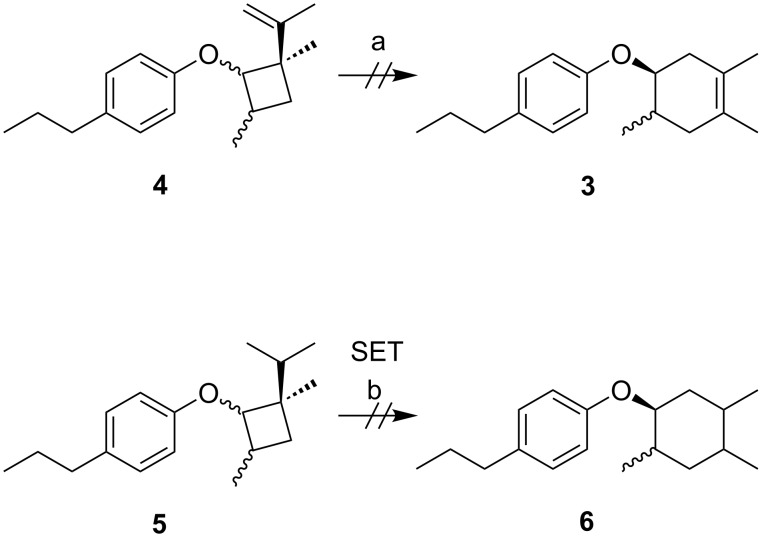
Unsuccessful rearrangement of cyclobutanes. Conditions: 1.0 M LiClO_4_/CH_3_NO_2_, carbon felt electrodes, a) no electricity, b)1.2 V vs Ag/AgCl, 0.1 F/mol.

When the reaction was carefully monitored by gas chromatography–mass spectrometry (GC–MS), we found that an adduct was generated in the early stage that was not the Diels–Alder product **3**. Therefore, we intentionally stopped the reaction after 0.1 F/mol of electricity had passed and attempted to identify the adduct. We found that it was a vinylcyclobutane **4**, whose formation was only observed in the early stage of the reaction and was completely consumed after 0.7 F/mol of electricity was passed (Scheme 4 and Figure 1). Vinylcyclobutanes are known to rearrange to cyclohexenes via thermal and/or photochemical processes and therefore, we carried out the anodic oxidation of vinylcyclobutane **4** in the absence of 2,3-dimethyl-1,3-butadiene (**2**, Scheme 5 and Supporting Information File 1, Figure S2). Interestingly, the rearrangement took place effectively to give Diels–Alder adduct **3** as an approximately *cis*/*trans* = 1:4 mixture, a significant difference from the synthetic outcome mentioned above (Scheme 3). Notably, no rearrangement was observed without electricity and the vinyl substituent was found to be essential for the transformation since no cyclohexane **6** was obtained from the cyclobutane **5** (Scheme 6). We also found that the Diels–Alder adduct **3** with an approximately *cis*/*trans* = 1:1 mixture was obtained in the early stage of the reaction (Supporting Information File 1, Figure S3).

**Scheme 7:**
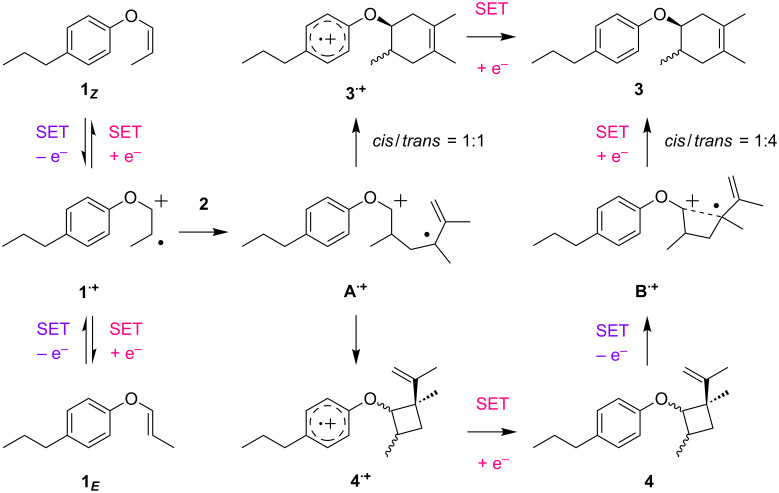
Proposed mechanism for the radical cation Diels–Alder reaction of aryl vinyl ether **1**.

Taken together, we can now propose a mechanism for the radical cation Diels–Alder reaction of the aryl vinyl ether **1** (Scheme 7). On the basis of the oxidation potentials, oxidative SET would selectively take place from the aryl vinyl ether **1** even in the presence of 2,3-dimethyl-1,3-butadiene (**2**) to generate the radical cation **1^·+^**, in which the stereochemistry derived from the starting material is lost. Indeed, the anodic oxidation of either the *Z*- (**1*_Z_***) or the *E*-form (**1*_E_***) in the absence of 2,3-dimethyl-1,3-butadiene (**2**) led to isomerization. In other words, inversion of the configuration of the radical cation **1^·+^** proceeds readily, leading to a loss in stereoselectivity. The radical cation **1^·+^** is then trapped by 2,3-dimethyl-1,3-butadiene (**2**) to construct the acyclic radical cation intermediate **A^·+^**, which is potentially converted into the aromatic radical cation with a six-membered ring (**3^·+^**) or a four-membered ring (**4^·+^**) via rapid intramolecular SET processes. It can be rationalized that the six-membered ring closure of the acyclic radical cation intermediate (**A^·+^**) would be a stepwise process, leading to the Diels–Alder adduct **3** as an approximately *cis*/*trans* = 1:1 mixture. However, since the oxidative SET-triggered rearrangement of the vinylcyclobutane **4** gives the Diels–Alder adduct **3** as an approximately *cis*/*trans* = 1:4 mixture, this should proceed via a different intermediate from the acyclic radical cation intermediate (**A^·+^**). Here, we propose the cyclic radical cation intermediate **B^·+^** for the rearrangement, imparting stereoselectivity for the six-membered ring closure. The anodic oxidation of the Diels–Alder adduct **3** does not give either the retro-reaction product **1** or the vinylcyclobutane **4** and therefore, it seems that the overall transformation involves irreversible steps. Thus, the radical cation Diels–Alder reaction of aryl vinyl ether **1** effectively proceeds either via “direct” or “indirect” pathways, affording the corresponding adduct **3** in excellent yield.

## Conclusion

In conclusion, we have demonstrated that the radical cation Diels–Alder reaction initiated by electrocatalysis in lithium perchlorate/nitromethane electrolyte solution is not limited to styrenes but was also effective for the aryl vinyl ether. While the mechanism is still controversial, most reported radical cation Diels–Alder reactions are highly stereoselective, even including stereospecific concerted examples. On the other hand, our current results clearly indicate that a stepwise mechanism is also effective for this class. Furthermore, the radical cation Diels–Alder reaction of the aryl vinyl ether is found to proceed via multiple unique pathways, including direct and indirect manners. Our findings described herein are beneficial to design novel SET-triggered cycloadditions, which are under development in our laboratory.

## Supporting Information

File 1 Additional scheme and figures, general remarks, synthesis and characterization data, including copies of ^1^H and ^13^C NMR spectra.
