# Supplementary material for: Stepwise radical cation Diels–Alder reaction via multiple pathways
Source: Beilstein J Org Chem. 2018 Mar 27;14:704–8. doi: 10.3762/bjoc.14.59 (PMC5905251; doi:10.3762/bjoc.14.59)

Supporting Information

for

Stepwise radical cation Diels–Alder reaction via multiple pathways

Ryo Shimizu[1], Yohei Okada[2] and Kazuhiro Chiba*[1]

Address: [1]Department of Applied Biological Science, Tokyo University of Agriculture and Technology, 3-5-8 Saiwai-cho, Fuchu, Tokyo 183-8509, Japan and [2]Department of Chemical Engineering, Tokyo University of Agriculture and Technology, 2-24-16 Naka-cho, Koganei, Tokyo 184-8588, Japan

Email: Kazuhiro Chiba - chiba@cc.tuat.ac.jp

Tel: (+81)-42-367-5667

Fax: (+81)-42-367-5667

*Corresponding author

Additional scheme and figures, general remarks, synthesis and characterization data, including copies of ^{1}H and ^{13}C NMR spectra

Table of contents

1. Additional scheme and figures p. S2

2. General remarks p. S4

3. Synthesis and characterization data p. S5

4. Copies of ^{1}H and ^{13}C NMR spectra p. S9

1. Additional scheme and figures

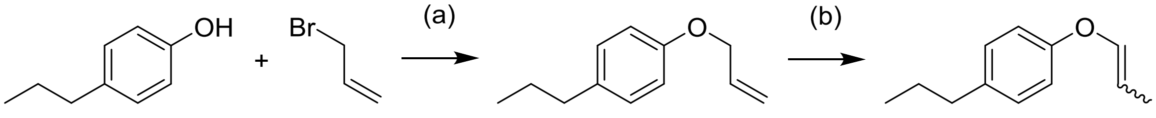

1

Conditions: (a) Allyl bromide, K_2CO_3, DMF. (b) *t*-BuOK, DMSO.

Scheme S1: Synthesis of the aryl vinyl ether **1**.

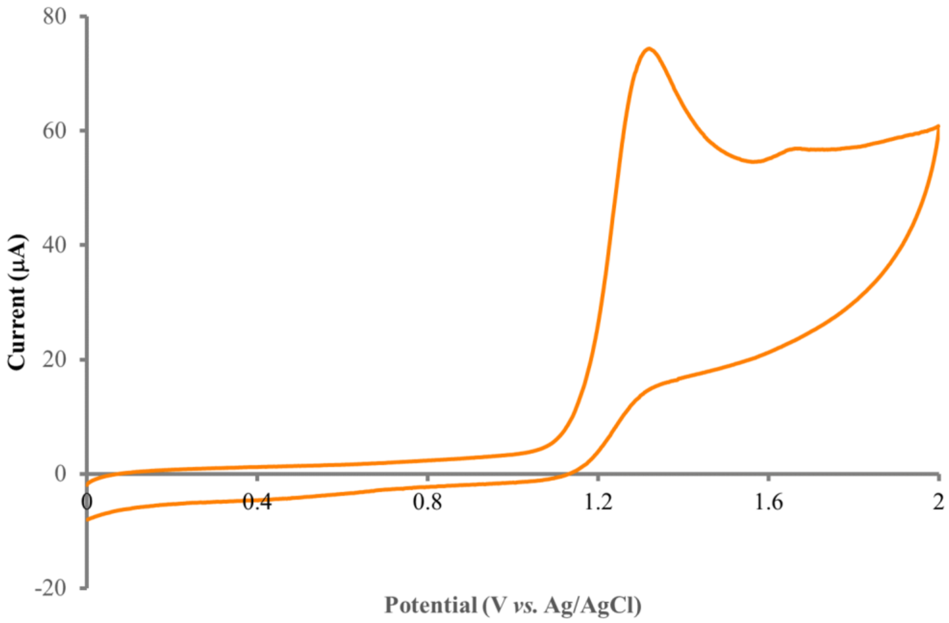

Figure S1: Cyclic voltammogram of the aryl vinyl ether **1**.

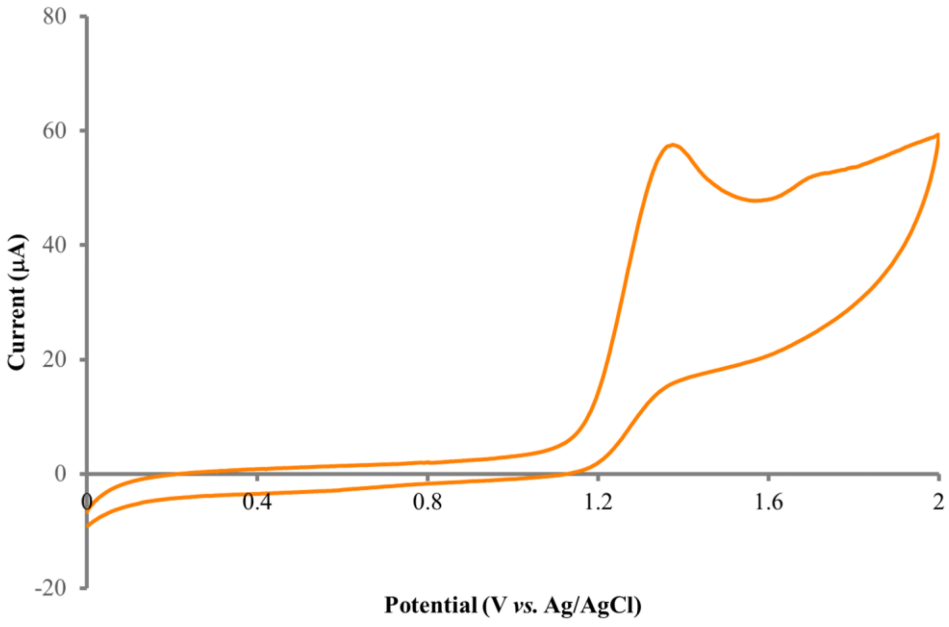

Figure S2: Cyclic voltammogram of the vinyl cyclobutane **4**.

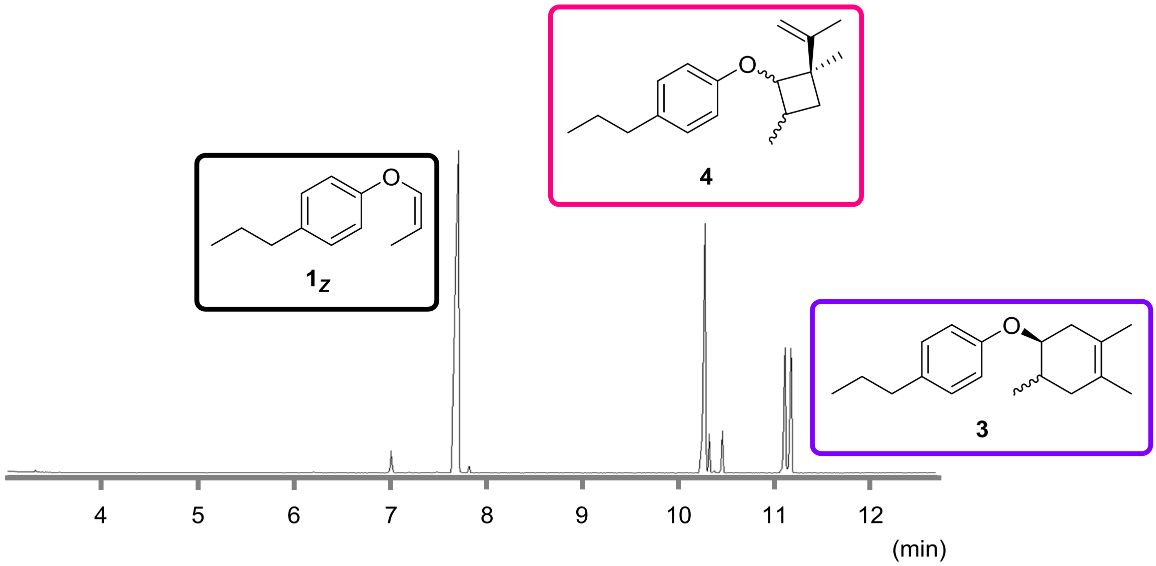

Figure S3: GC–MS Profile of the Reaction at 0.1 F/mol.

2. General remarks

All reagents and solvents were purchased from commercial sources and used without further purification. Reactions were monitored by thin-layer chromatography (TLC) carried out on silica gel plates, with detection by UV absorption (254 nm) and by heating the plates after dipping them in a solution of 12 molybdo(VI) phosphoric acid *n*-hydrate in 95% ethanol. Silica gel (particle size 40−50 μm) was used for column chromatography. ^{1}H NMR spectra were collected on 600 or 400 MHz NMR spectrometers using the deuterated solvent as an internal deuterium reference. Chemical shift data are given in δ units calibrated with residual protic solvent. The multiplicity of a signal is indicated as follows: s, singlet; d, doublet; t, triplet; q, quartet; quin, quintet; m, multiplet. ^{13}C NMR spectra were collected on 150 or 100 MHz spectrometers with proton decoupling using the deuterated solvent as an internal carbon reference.

3. Synthesis and Characterization Data

*(Z)-1-(Prop-1-en-1-yloxy)-4-propylbenzene (**1**$_Z$)*

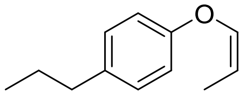

1$_Z$

To a solution of 4-propylphenol in DMF, allyl bromide (3.0 equiv.) and K_2CO_3 (4.5 equiv.) were added. The resulting reaction mixture was stirred at 70 °C until completion of the reaction (determined by TLC), followed by dilution with EtOAc. The organic layer was washed with brine, dried over $MgSO_4$, and concentrated in vacuo. Purification by silica gel column chromatography (Hex/EtOAc) gave the desired allylated compounds. To a solution of the resulting allylated compound in DMSO, *t*-BuOK (8.0 equiv) was added. The resulting reaction mixture was stirred at room temperature for 6 h, followed by dilution with Hex. The layers were separated, the DMSO layer was extracted several times with Hex, and the combined Hex phases were washed with brine, dried over $MgSO_4$, and concentrated in vacuo. Purification by silica gel column chromatography (Hex/EtOAc) gave the desired aryl vinyl ether. ^{1}H NMR (600 MHz, $CDCl_3$) δ 7.10 (2H, d, *J* = 8.7 Hz), 6.90 (2H, d, *J* = 8.7 Hz), 6.35 (1H, dq, *J* = 5.9, 2.1 Hz), 4.84 (1H, dq, *J* = 6.8, 6.8 Hz), 2.54 (2H, t, *J* = 7.3 Hz), 1.71 (3H, dd, *J* = 6.8, 2.1 Hz), 1.61 (2H, sext, *J* = 7.3 Hz), 0.93 (3H, t, *J* = 7.3 Hz); ^{13}C NMR (150 MHz, $CDCl_3$) δ 155.7, 141.3, 136.7, 129.5, 116.1, 106.9, 37.3, 24.8, 13.8, 9.4.

*(E)-1-(Prop-1-en-1-yloxy)-4-propylbenzene (**1**$_E$)*

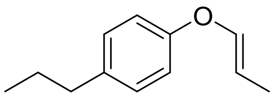

1$_E$

^{1}H NMR (600 MHz, $CDCl_3$) δ 7.10 (2H, d, *J* = 8.7 Hz), 6.89 (2H, d, *J* = 8.7 Hz), 6.40 (1H, dq, *J* = 12.1, 1.9 Hz), 5.33 (1H, dq, *J* = 12.4, 6.9 Hz), 2.54 (2H, t, *J* = 7.3 Hz), 1.66 (3H, dd, *J* = 6.9, 1.9 Hz), 1.61 (2H, sext, *J* = 7.3 Hz), 0.93 (3H, t, *J* = 7.3 Hz); ^{13}C NMR (150 MHz, $CDCl_3$) δ 155.5, 142.5, 136.8, 129.5, 116.2, 107.7, 37.3, 24.8, 13.8, 12.4.

*1-Propyl-4-((3,4,6-trimethylcyclohex-3-en-1-yl)oxy)benzene (**3**)*

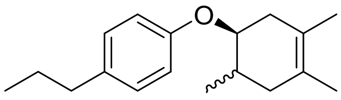

3

To a solution of lithium perchlorate (1.06 g, 10.0 mmol) in nitromethane (10 mL) stirred at rt was added 1-(prop-1-en-1-yloxy)-4-propylbenzene (**1**, 148 µL, 0.8 mmol) and 2,3-dimethyl-1,3-butadiene (**2**, 180 µL, 1.6 mmol). The resulting reaction mixture was electrolyzed at r.t. using carbon felt electrodes (2 cm × 2 cm) in an undivided cell with stirring at a constant potential of 1.2 V vs Ag/AgCl. After completion (monitored by TLC and GC–MS), the reaction mixture was diluted with EtOAc and washed with brine. The organic layer was dried over $MgSO_4$, filtered, and concentrated in vacuo to give a crude product as a yellow oil. Silica gel column chromatography (EtOAc/Hex = 1:150) gave the title compound as a colorless oil. *cis*: ^{1}H NMR ($CDCl_3$, 600 MHz) δ 7.05 (2H, d, *J* = 8.9 Hz), 6.82 (2H, d, *J* = 8.9 Hz), 4.38 (1 H, td, *J* = 5.5, 2.8 Hz), 2.51 (2H, t, *J* = 7.6 Hz), 2.24-2.21 (2H, m), 2.11-2.03 (2H, m), 2.02-1.95 (1H, m), 1.64-1.57 (8H, m), 0.99 (3H, d, *J* = 6.9 Hz), 0.93 (3H, t, *J* = 7.6 Hz); ^{13}C NMR (150 MHz, $CDCl_3$) δ 156.5, 134.7, 129.3, 124.6, 121.8, 115.9, 76.0, 37.3, 36.9, 35.0, 31.5, 24.9, 19.1, 18.6, 15.4, 13.9. *trans*: ^{1}H NMR (600 MHz, $CDCl_3$) δ 7.07 (2H, d, *J* = 8.2 Hz), 6.85 (2H, d, *J* = 8.2 Hz), 4.05 (1H, td, *J* = 8.6, 5.5 Hz), 2.51 (2H, t, *J* = 7.6 Hz), 2.41 (1H, dd, *J* = 17.2, 4.8 Hz), 2.18 (1H, dd, *J* = 17.2, 4.8 Hz), 2.07 (1H, dd, *J* = 16.5, 7.6 Hz), 2.03-1.95 (1H, m),

1.84-1.76 (1H, m), 1.64-1.57 (8H, m), 1.04 (3H, d, *J* = 6.9 Hz), 0.93 (3H, t, *J* = 7.6 Hz); ^{13}C NMR (150 MHz, $CDCl_3$) δ 156.7, 134.8, 129.4, 125.0, 122.9, 115.9, 79.0, 39.5, 37.3, 37.0, 34.1, 24.9, 18.9, 18.6, 17.9, 14.0; HRMS $[M + H]^+$ calculated for $C_{18}H_{27}O$ 259.2062, found 259.2054.

*1-(2,4-Dimethyl-2-(prop-1-en-2-yl)cyclobutoxy)-4-propylbenzene (**4**)*

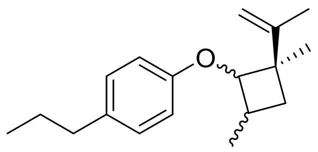

4

To a solution of lithium perchlorate (1.06 g, 10.0 mmol) in nitromethane (10mL) stirred at rt was added 1-(prop-1-en-1-yloxy)-4-propylbenzene (**1**) (148 μL, 0.8 mmol) and 2,3-dimethyl-1,3-butadiene (**2**) (180 μL, 1.6 mmol). The resulting reaction mixture was electrolyzed at rt using carbon felt electrodes (2 cm × 2 cm) in an undivided cell with stirring at a constant potential of 1.2 V vs. Ag/AgCl. After the passage of 0.1 F/mol, the reaction mixture was diluted with EtOAc and washed with brine. The organic layer was dried over $MgSO_4$, filtered, and concentrated in vacuo to give a crude product as a yellow oil. Silica gel column chromatography (EtOAc/Hex = 1:150) gave the title compound as a colorless oil. *One Diastereomer*: ^{1}H NMR (600 MHz, $CDCl_3$) δ 7.05 (2H, d, *J* = 8.2 Hz), 6.83 (2H, d, *J* = 8.2 Hz), 4.68 (1H, t, *J* = 1.4 Hz), 4.63 (1H, s), 4.12 (1H, d, *J* = 7.3 Hz), 2.51 (2H, t, *J* = 7.3 Hz), 2.44-2.36 (1H, m), 1.83 (1H, t, *J* = 10.1 Hz), 1.67 (3H, s), 1.60 (2H, sext, *J* = 7.3 Hz), 1.36 (1H, t, *J* = 10.1 Hz), 1.32 (3H, s), 1.19 (3H, d, *J* = 6.4 Hz), 0.92 (3H, t, *J* = 7.3 Hz); ^{13}C NMR (150 MHz, $CDCl_3$) δ 156.6, 152.6, 135.2, 129.3, 116.3, 108.0, 83.9, 47.2, 37.3, 33.8, 33.5, 24.8, 20.2, 19.8, 18.9, 13.9; HRMS $[M + H]^+$ calculated for $C_{18}H_{27}O$ 259.2062, found 259.2059.

To a solution of lithium perchlorate (1.06 g, 10.0 mmol) in nitromethane (10mL) stirred at r.t. was added 1-(prop-1-en-1-yloxy)-4-propylbenzene (**1**, 148 μL, 0.8 mmol) and 2,3-dimethyl-1-butene (1.98mL, 16 mmol). The resulting reaction mixture was electrolyzed at r.t. using carbon felt electrodes (2 cm × 2 cm) in an undivided cell with stirring at a constant potential of 1.2 V vs. Ag/AgCl. After the passage of 0.8 F/mol, the reaction mixture was diluted with EtOAc and washed with brine. The organic layer was dried over $MgSO_4$, filtered, and concentrated in vacuo to give a crude product as a yellow oil. Silica gel column chromatography (EtOAc : Hex = 1 : 150) gave the title compound as a colorless oil. *One Diastereomer*: ^{1}H NMR (600 MHz, $CDCl_3$) δ 7.06 (2H, d, *J* = 8.3 Hz), 6.78 (2H, d, *J* = 8.3 Hz), 3.93 (1H, d, *J* = 7.6 Hz), 2.51 (2H, t, *J* = 7.6 Hz), 2.38-2.31 (1H, m), 2.09 (1H, quin, *J* = 6.9 Hz), 2.01 (1H, t, *J* = 10.6 Hz), 1.60 (2H, sext, *J* = 7.6 Hz), 1.15 (3H, d, *J* = 6.9 Hz), 1.13 (3H, s), 0.94-0.91 (4H, m), 0.87 (3H, d, *J* = 6.9 Hz), 0.84 (3H, d, *J* = 6.9 Hz); ^{13}C NMR (150 MHz, $CDCl_3$) δ 157.2, 134.6, 129.3, 115.3, 87.2, 45.1, 37.3, 33.6, 33.3, 30.1, 24.9, 22.5, 20.0, 18.7, 17.9, 13.4; HRMS $[M + H]^+$ calculated for $C_{18}H_{29}O$: 261.2218, found: 261.2225.

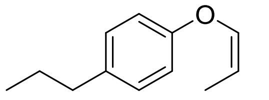

1*Z*

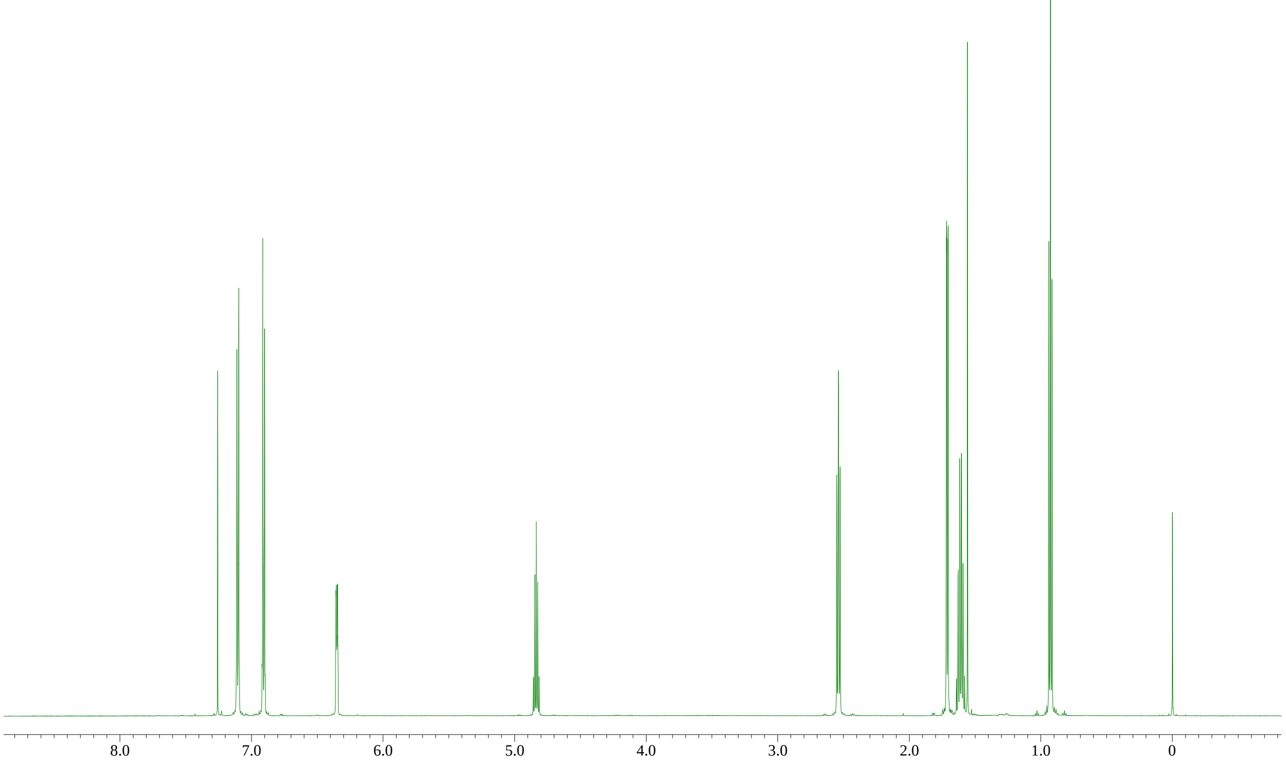
8.0
7.0
6.0
5.0
4.0
3.0
2.0
1.0
0

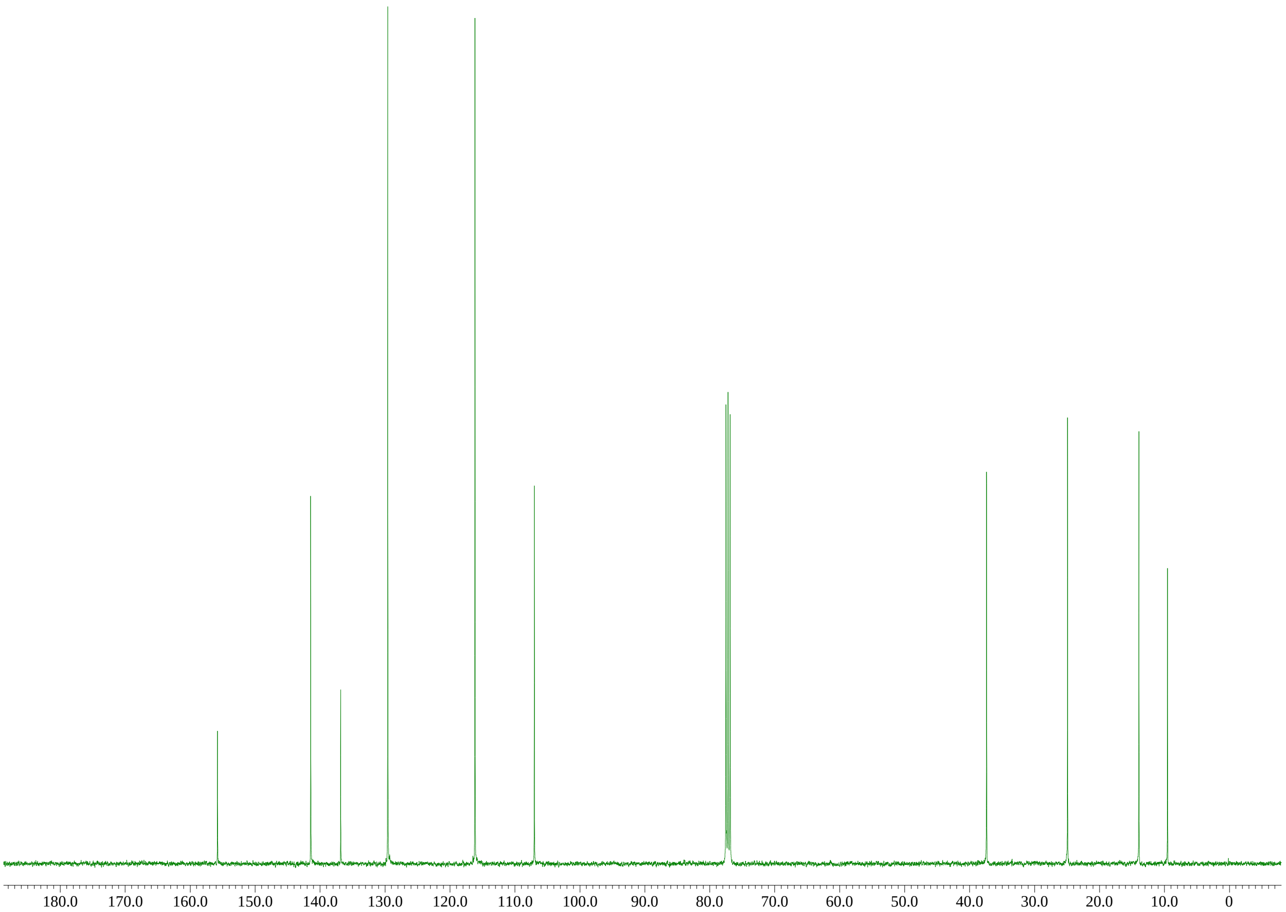
180.0
170.0
160.0
150.0
140.0
130.0
120.0
110.0
100.0
90.0
80.0
70.0
60.0
50.0
40.0
30.0
20.0
10.0
0

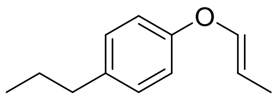

$\mathbf{1}_{E}$

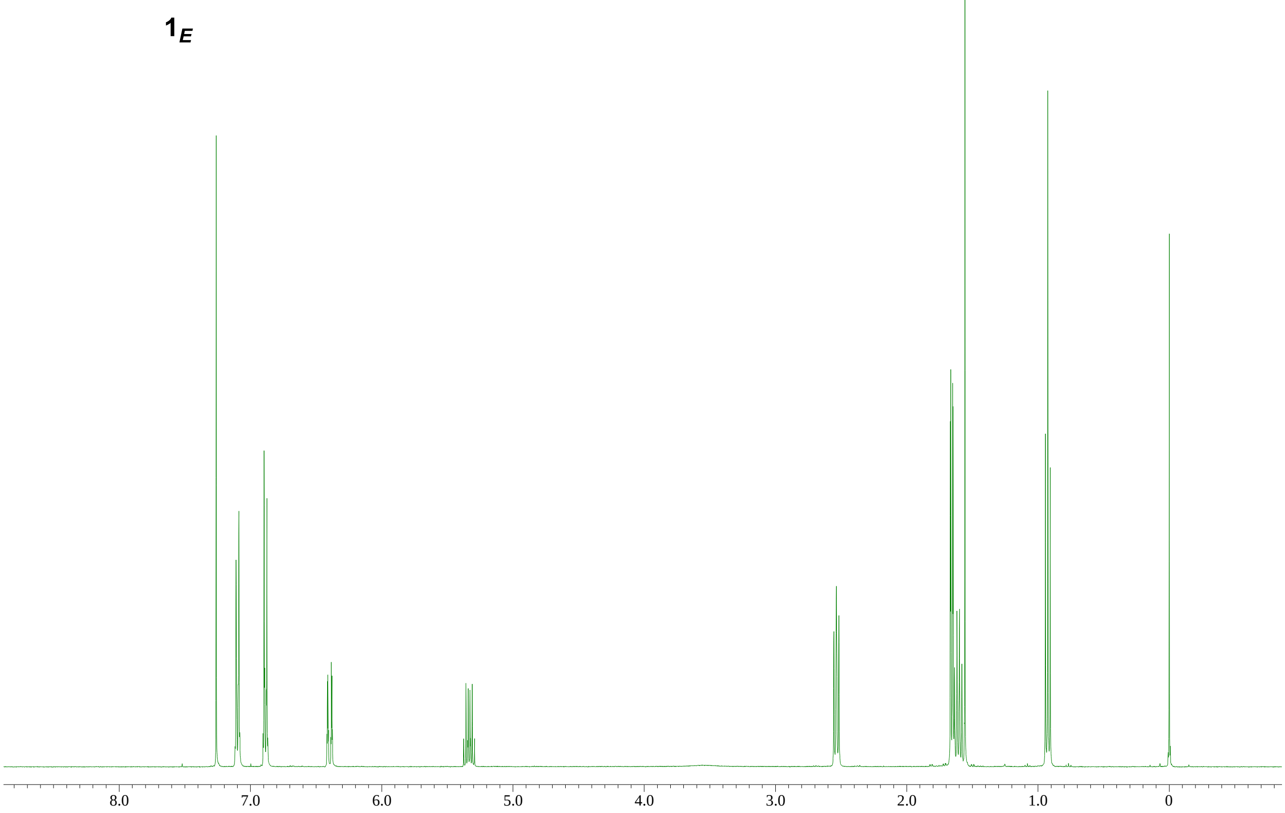
8.0 7.0 6.0 5.0 4.0 3.0 2.0 1.0 0

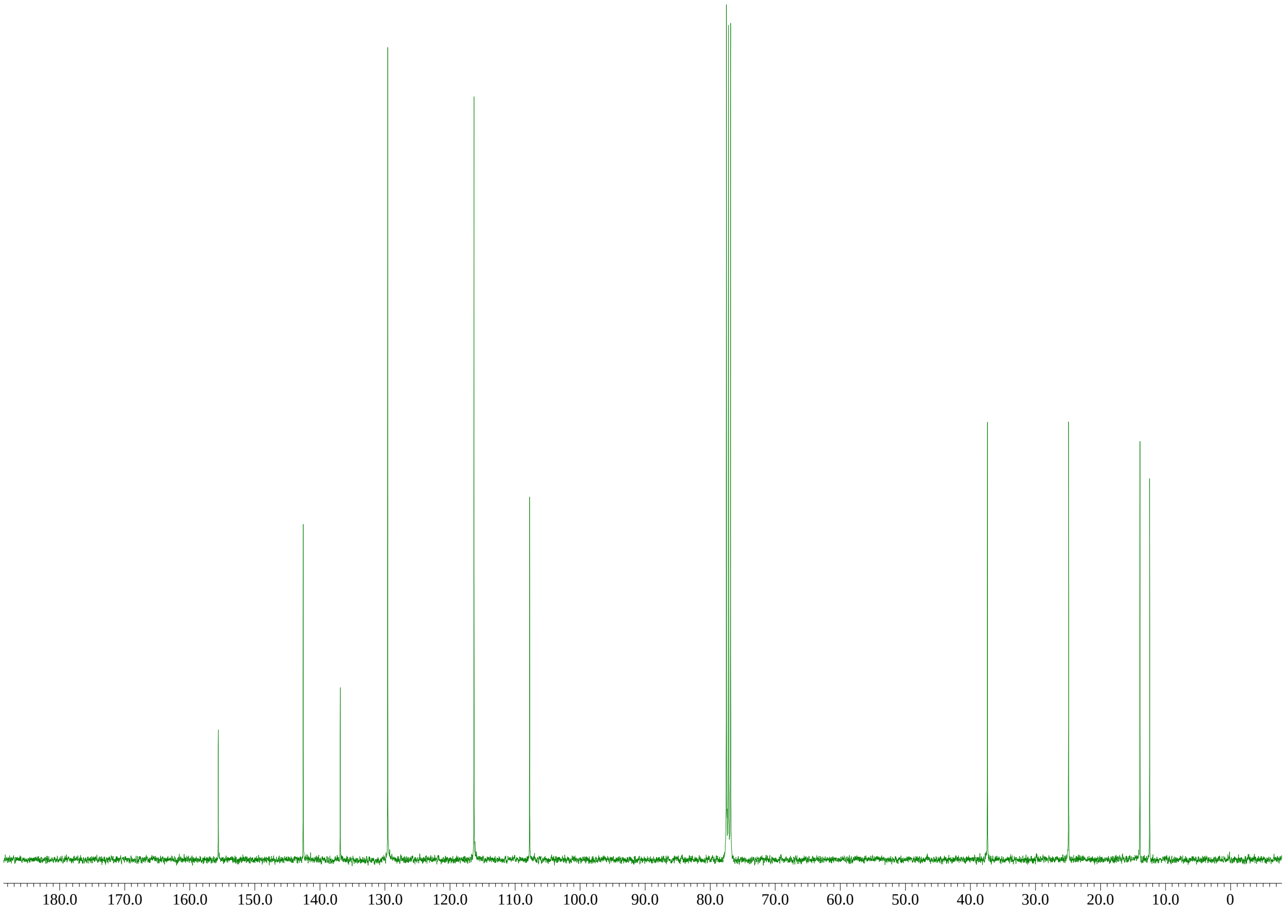
180.0 170.0 160.0 150.0 140.0 130.0 120.0 110.0 100.0 90.0 80.0 70.0 60.0 50.0 40.0 30.0 20.0 10.0 0

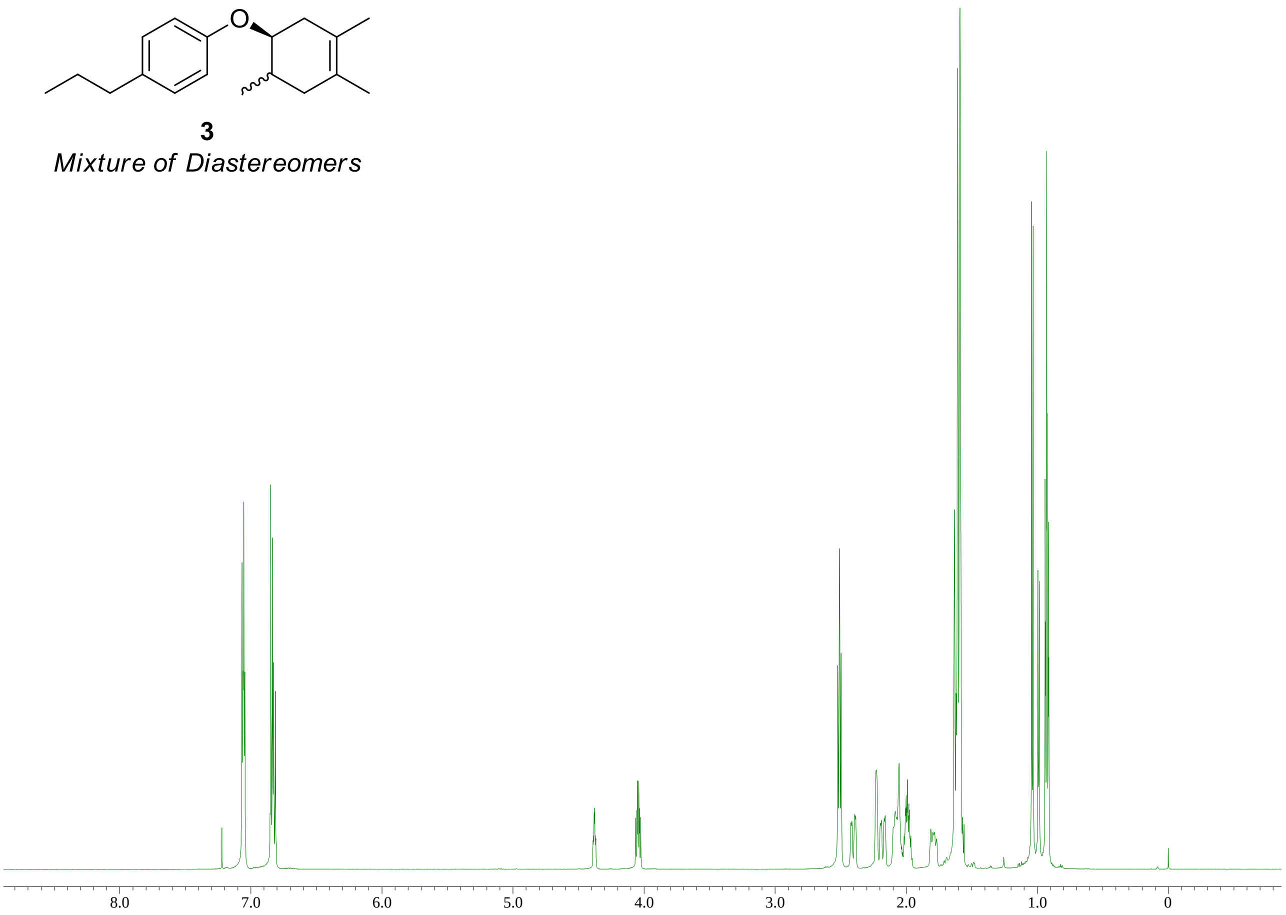
3
Mixture of Diastereomers
8.0
7.0
6.0
5.0
4.0
3.0
2.0
1.0
0

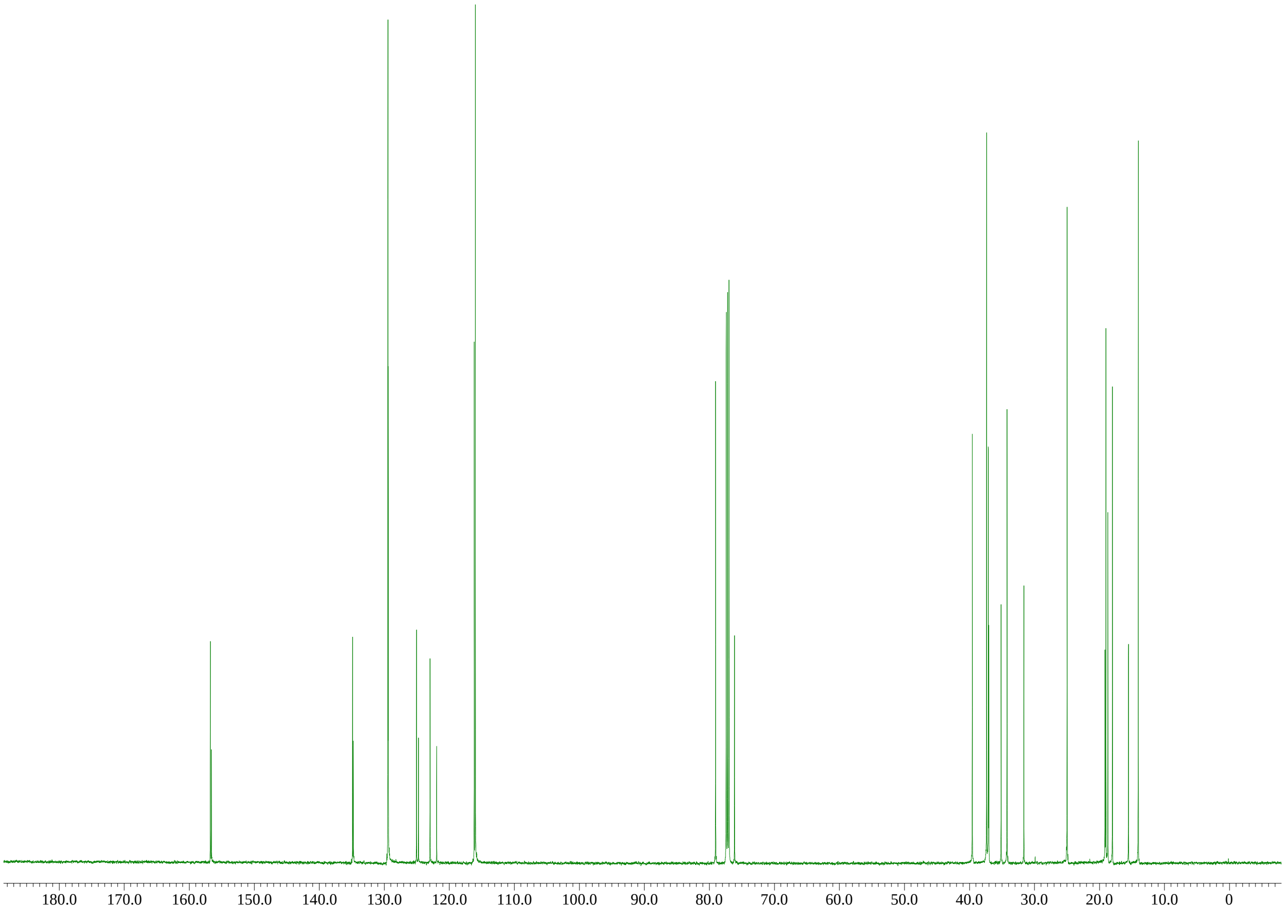
180.0
170.0
160.0
150.0
140.0
130.0
120.0
110.0
100.0
90.0
80.0
70.0
60.0
50.0
40.0
30.0
20.0
10.0
0

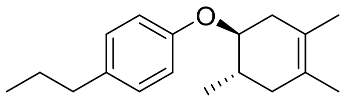

$\mathbf{3}_t$

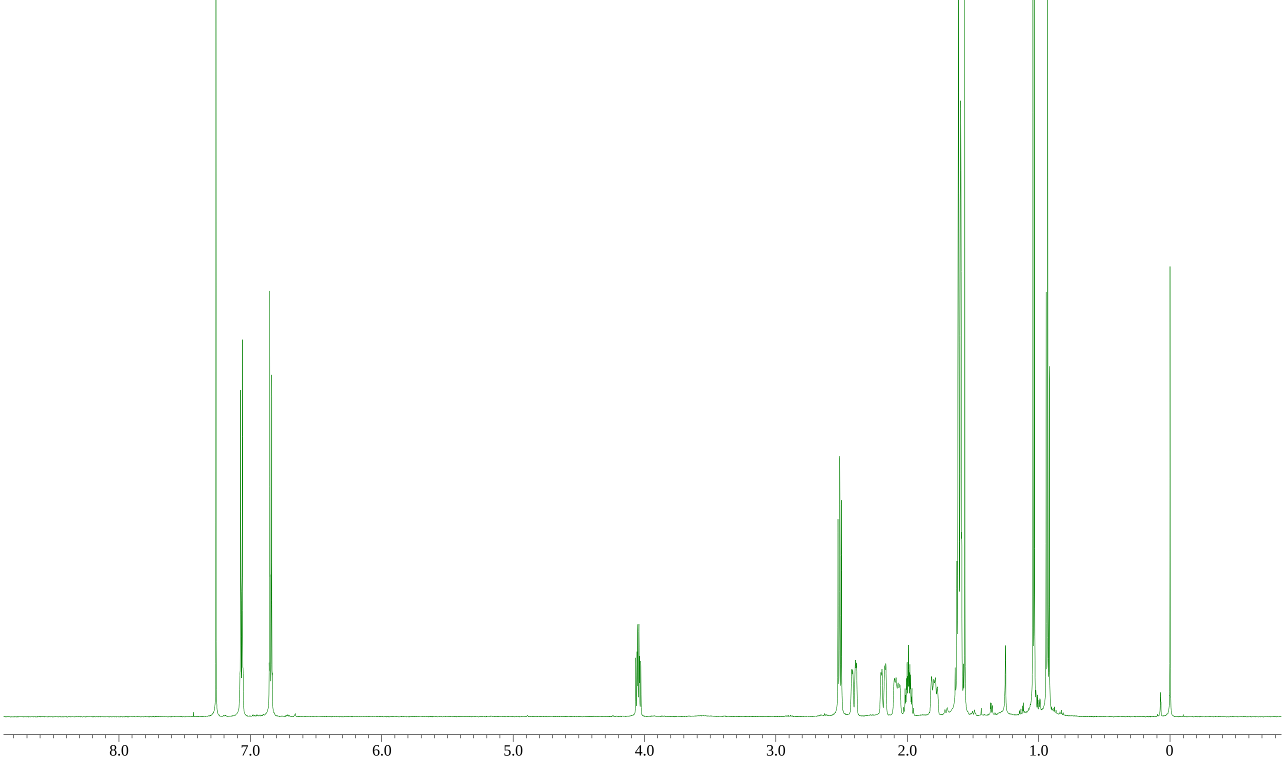

8.0
7.0
6.0
5.0
4.0
3.0
2.0
1.0
0

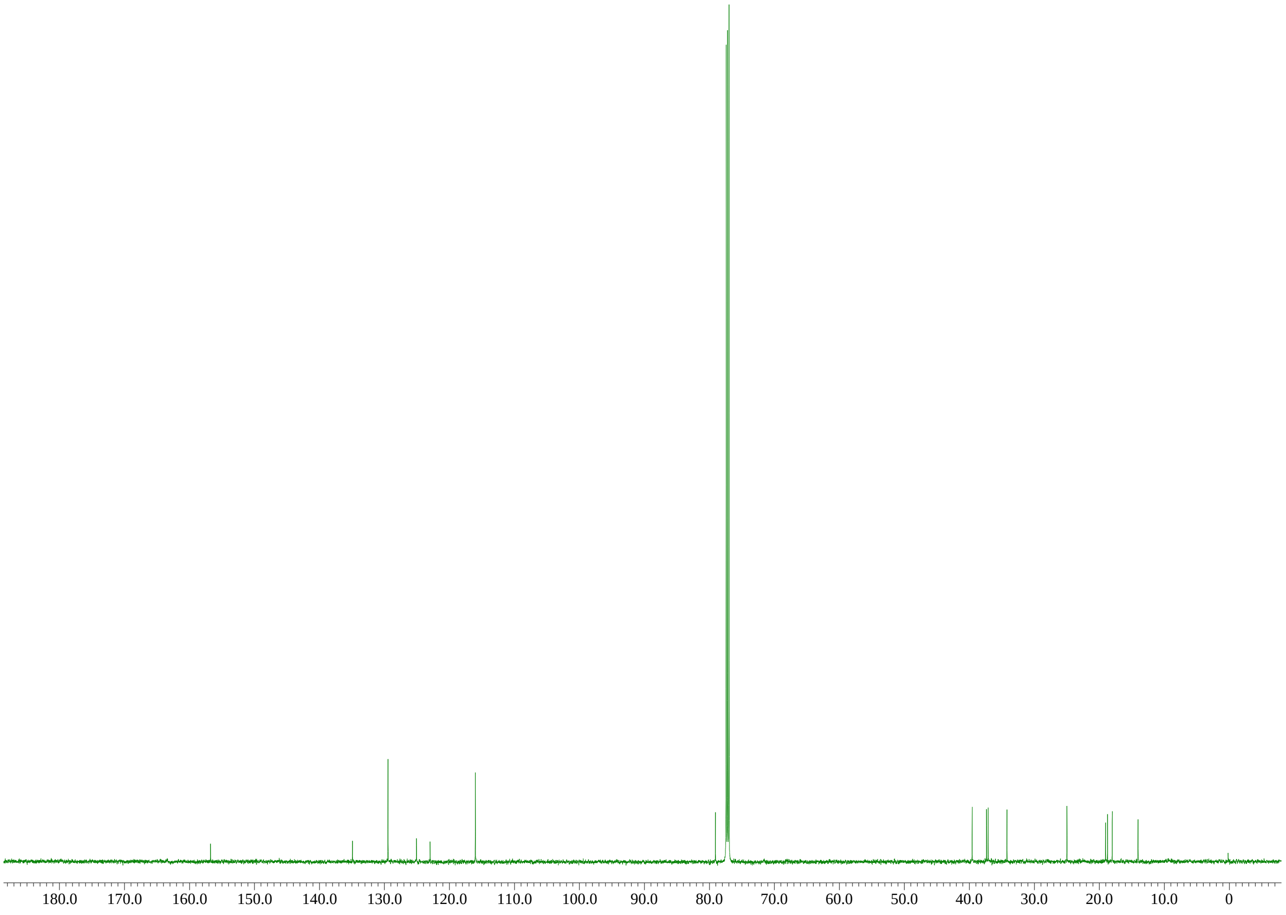

180.0
170.0
160.0
150.0
140.0
130.0
120.0
110.0
100.0
90.0
80.0
70.0
60.0
50.0
40.0
30.0
20.0
10.0
0

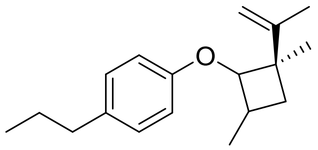

4
One Diastereomer

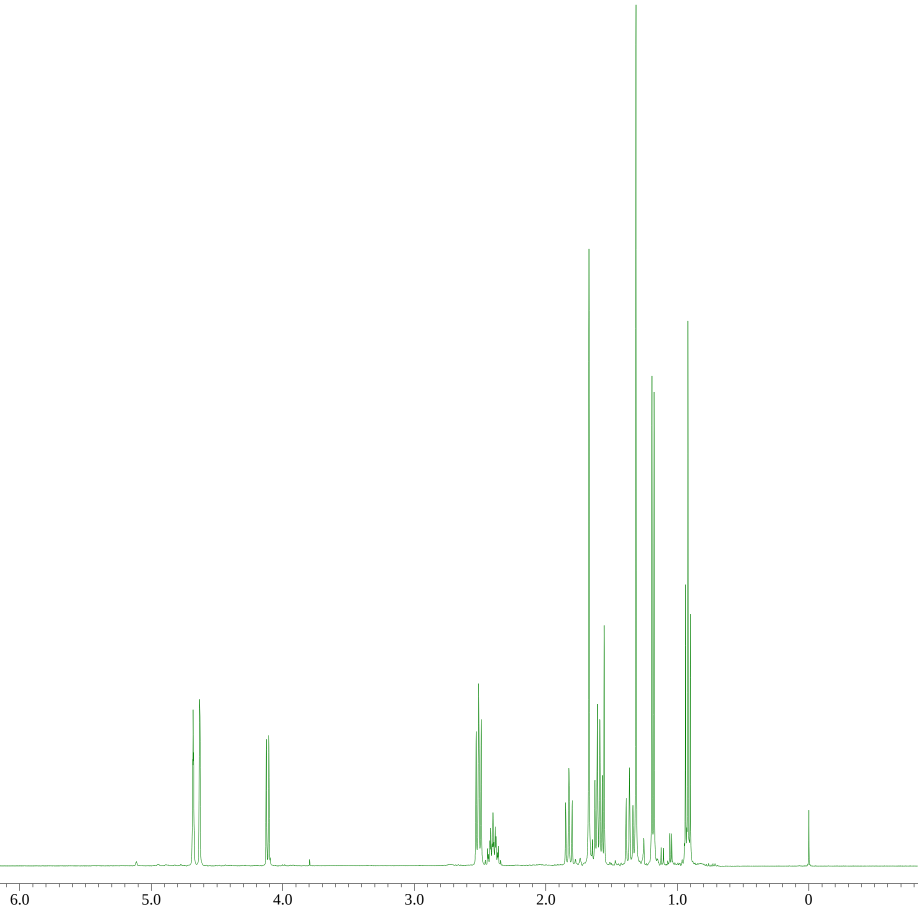

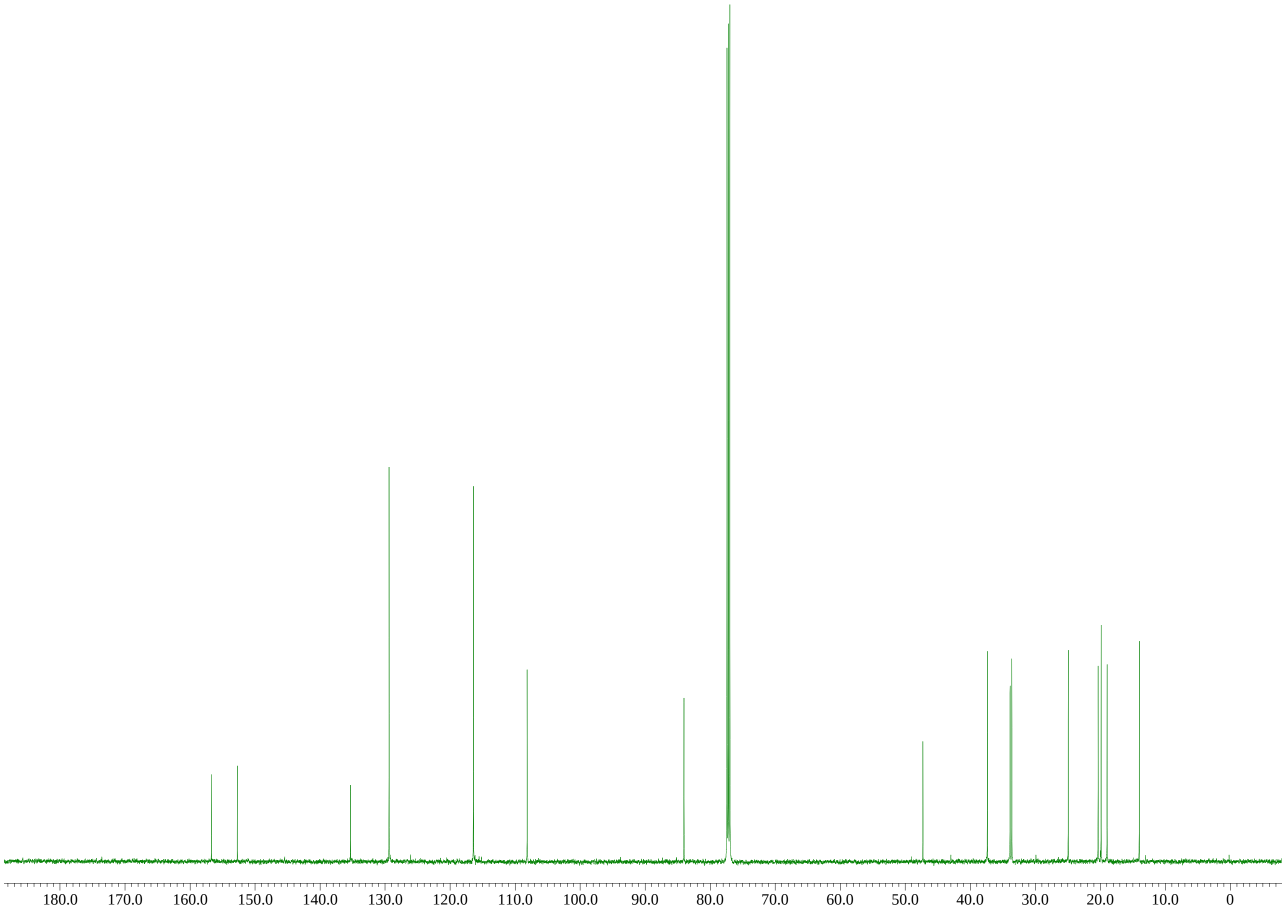

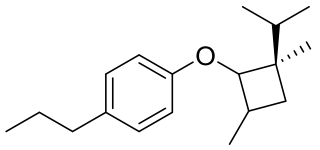

5
One Diastereomer

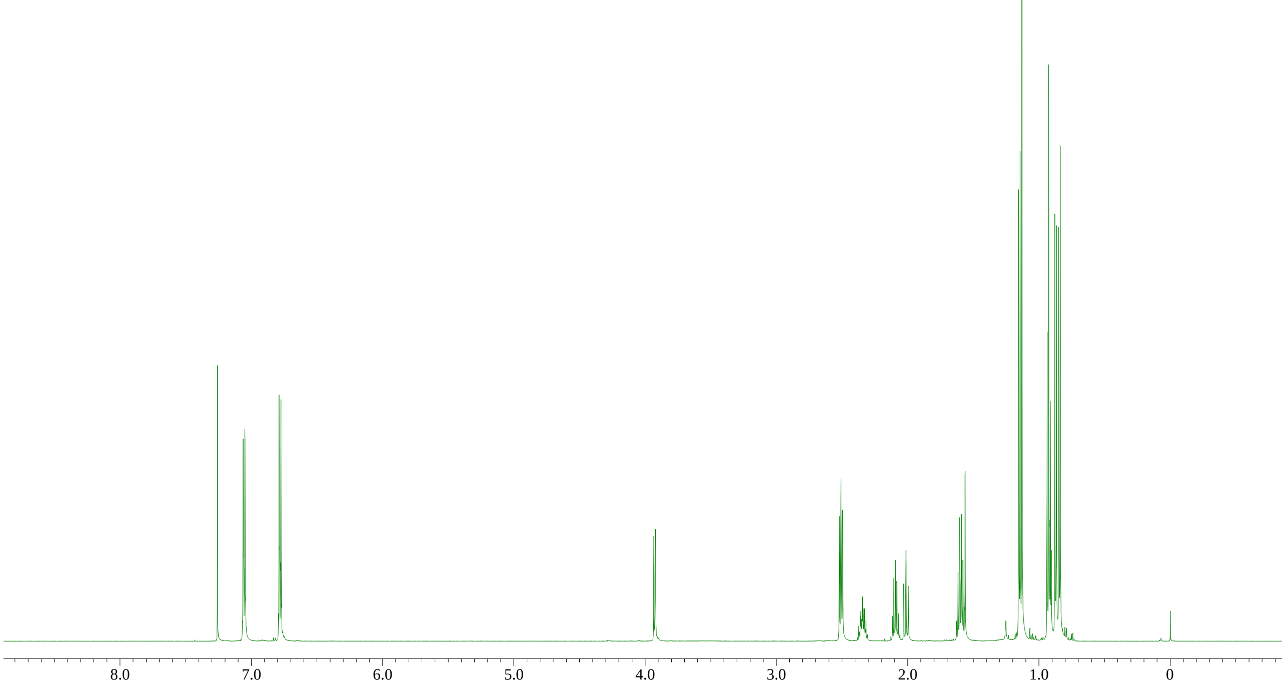

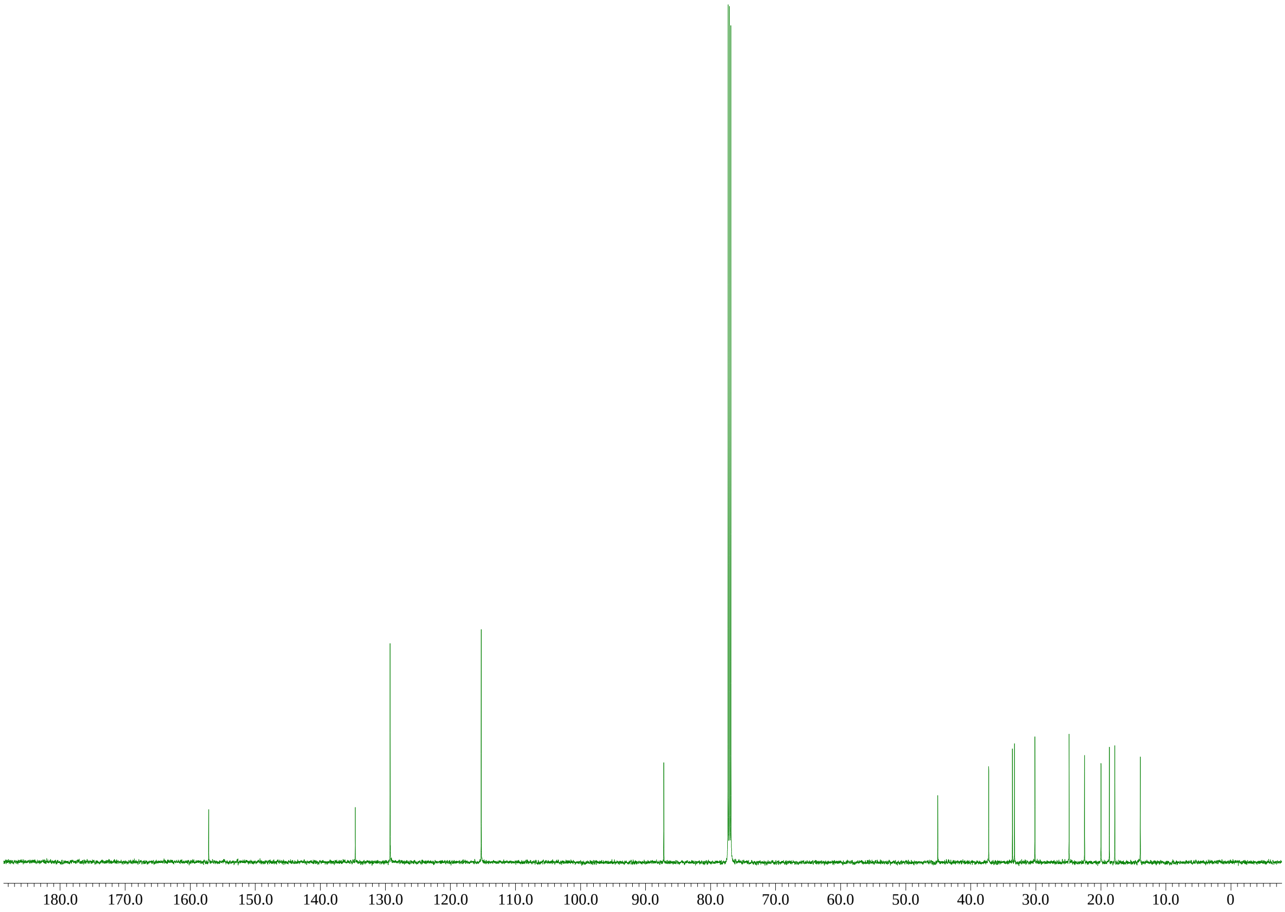

Supplement: File 1 — Additional scheme and figures, general remarks, synthesis and characterization data, including copies of 1H and 13C NMR spectra. [file Beilstein_J_Org_Chem-14-704-s001.pdf]
